# Ni nanoparticle supported reduced graphene oxide as a highly active and durable heterogeneous material for coupling reactions†

**DOI:** 10.1039/c8na00245b

**Published:** 2019-01-29

**Authors:** Surjyakanta Rana, G. Bishwa Bidita Varadwaj, S. B. Jonnalagadda

**Affiliations:** School of Chemistry & Physics, College of Agriculture, Engineering & Science, University of KwaZulu-Natal Durban South Africa jonnalagaddas@ukzn.ac.za surjya.nou@gmail.com +27 31 260 3091 +27 31 260 7325 ext. 3090

## Abstract

We report the loading of highly air stable Ni(0) nanoparticles (average particle size = 11 nm) on the surface of a reduced graphene oxide (RGO) material. The material was characterized using different techniques, including Raman spectroscopy, XRD, TEM, SEM, and HRTEM analysis. The Ni(0)@RGO catalyst showed superb efficiency towards Kumada–Corriu C–C cross-coupling reactions, with 92% yield of 4-methoxybiphenyl at 60 °C. The recycled material can be reused up to the 5^th^ cycle after regeneration by calcination, without loss of activity.

## Abstract 2

Ni nanoparticle supported reduced graphene oxide is shown to be a highly active and durable heterogeneous catalyst for Kumada–Corriu cross-coupling reactions.

## Graphical abstract

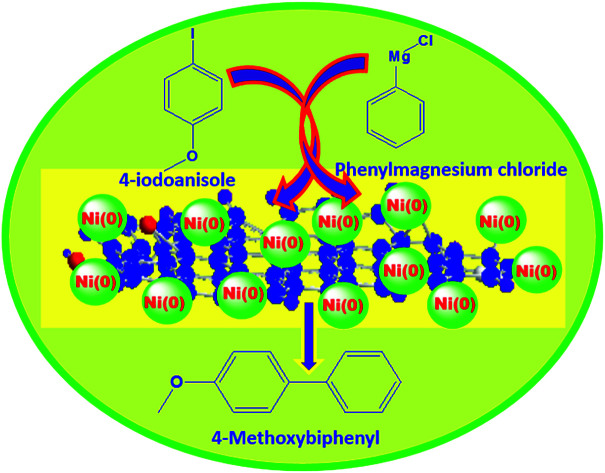

Over the last few years, carbon-based reduced graphene oxide (RGO) has attracted the attention of many researchers, particularly in the field of catalysis.^1^ Due to its exceptional characteristics, such as its high surface area and electronic properties, reduced graphene oxide has opened up new opportunities for its use as a next generation catalyst and catalyst support material.^2–9^ The stable dispersion of reduced graphene oxide both in aqueous and organic solvents makes it more ideal as a catalyst support. Generally, in order to enhance the efficiency of conversions in various reactions, different metal nanoparticles loaded on a reduced graphene oxide support have been explored. Graphene oxide can be synthesized by different graphite to sodium nitrate ratio. Out of different ratio of graphite to sodium nitrate materials, a 2 : 1 ratio increases the proportion of functional groups on the surface of the graphene oxide material. These functional groups allow for the growth of the metal nanoparticles on the surface.^10^ Nanoparticles have been explored for various applications due to their high surface to volume ratio.^11–16^ A survey of the literature shows the use of various metal nanoparticles, such as Au, Pt, and Pd supported RGO, as catalysts for different organic reactions.^17–22^ Most of these studies used noble metal supported reduced graphene oxide-based materials as catalysts, although only a few have employed low cost metal supported reduced graphene oxide-based materials for selective organic transformations.^23–26^

Broadly speaking, C–C cross-coupling reactions have been widely used for the preparation of different pharmaceutical products, molecular organic materials, and bioactive compounds.^27–29^ Cross-coupling reactions are one of the most significant C–C bond-forming reactions in organic synthesis. This reaction provides impressive results when using Pd complexes with phosphine ligands, or Pd as a ligand-free homogeneous catalyst. Homogeneous phosphine complexes are required to be handled under an inert atmosphere because they are more sensitive to moisture and exposure to air. When a ligand-free homogeneous catalyst is used, its recovery after reaction is very difficult. Therefore, for sustainability purposes, efficient reusable heterogeneous catalysts that can replace homogenous catalysts for cross-coupling reactions are of great significance. Hence, various heterogeneous Pd nanoparticle supported graphene oxide and functional graphene oxide materials have been developed as catalysts for coupling reactions.^30–32^ Some of these reported Pd-based catalysts are highly efficient and give high yields of the coupling product in the presence of different additives. However, the literature shows very few reports using Ni nanoparticle-based RGO catalysts for C–C coupling reactions.

In this communication, we report the novel synthesis of a Ni(0) modified reduced graphene oxide nanocomposite [Ni(0)RGO] material. During preparation, a 2 : 1 ratio of graphite to sodium nitrate was used for the synthesis of the support material. Without the need for any additives, it was capable of catalysing C–C coupling reactions with excellent yields.

The X-ray diffraction spectra of (a) GO and (b) Ni(0)RGO are illustrated in Fig. 1. In Fig. 1(a), the spectrum shows 2*θ* ≈ 10.74, corresponding to the (002) plane of GO.^4,33,34^ In Ni-modified material (Fig. 1(b)), the high intensity peak of the (002) plane of graphene oxide is shifted towards a higher degree, and appears as a broad peak at 2*θ* ≈ 25, suggesting that the graphene oxide is completely reduced to formed reduced graphene oxide (RGO).

**Fig. 1:**
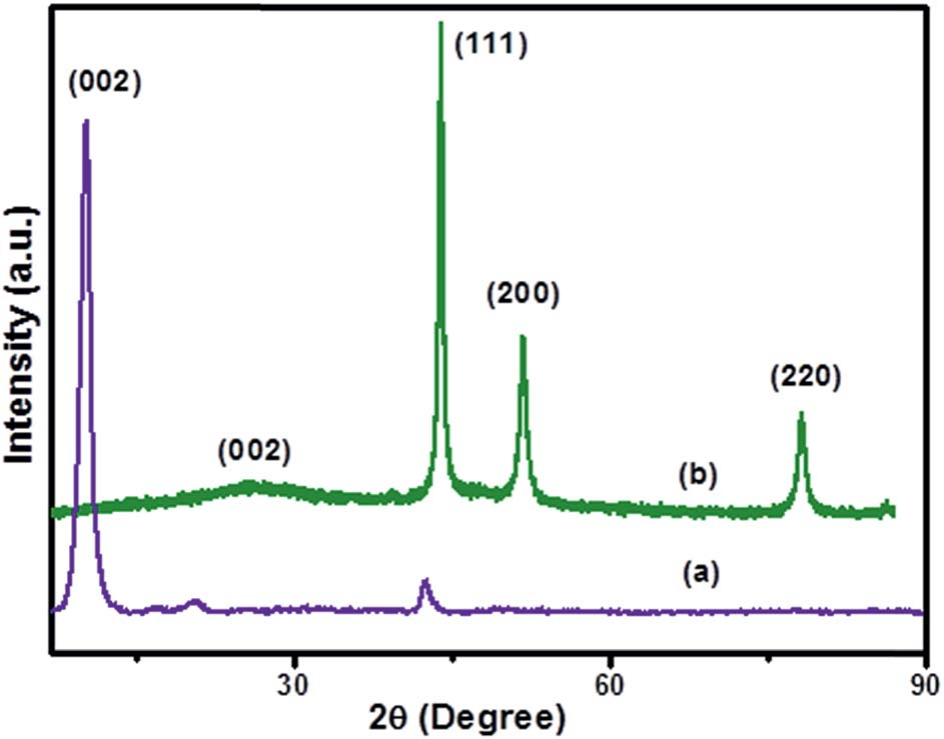
X-ray powder diffraction spectra of (a) GO and (b) Ni(0)RGO samples.

In Fig. 1(b), the peaks at 2*θ* ≈ 44.79, 52.24, and 78.2 correspond to the (111), (200), and (220) planes of the Ni metal nanoparticles (JCPDS card no. 01-071-4655). The average crystallite size of the synthesized nanomaterial was 11.35 nm, which is calculated from the Scherrer equation. The XRD pattern of Fig. 1(b) gives further confirmation of the absence of Ni(OH)_2_ and NiO peaks in the Ni(0)RGO sample.

Raman spectra of the (a) GO and (b) Ni(0)RGO catalysts are shown in ESI Fig. S1.† The Raman spectra give information about the disorder in the sp^2^-hybridized carbon atoms and σ-sp^2^ bonded carbon atoms, which correspond to the D band and G band, respectively. In case of graphene oxide material, the peaks at 1350 cm^−1^ and 1590 cm^−1^ represent the D band and G band, and the intensity of the *I*_d_/*I*_g_ ratio is 0.63. Following the modification of Ni metal on the graphene oxide surface to form Ni nanoparticles on reduced graphene oxide, the peak positions of the D and G bands remain constant. However, there is an increase in the intensity of the *I*_d_/*I*_g_ ratio from 0.63 to 0.67.

SEM images of the (a) GO and (b) Ni(0)RGO samples are shown in ESI Fig. S2.† The SEM images give confirmation of the layered structure of graphene oxide. The SEM/EDX and color mapping images of the Ni(0)RGO sample are illustrated in ESI Fig. S3.† This technique gives information about the type of elements present on the material. In this color mapping image, elements like C, Ni, and O are present on the catalyst surface, and are indicated by different colors.

TEM images and electron diffraction patterns of the Ni nanoparticles are shown in Fig. 2. The TEM images provide information about the Ni nanoparticles. The electron diffraction pattern of the three planes, (111), (200), and (220), clearly shows the features of the Ni particles, which resemble the *d*-spacing of the Ni phases as obtained from the powder X-ray diffraction study. A particle size histogram of the Ni nanoparticles is shown in ESI Fig. S4.† From this histogram, we calculated the average particle size of the Ni nanoparticles as about 11 nm, and these particles were present on the surface of the reduced graphene material. A high resolution image (HRTEM) of the Ni(0)RGO catalyst is shown in ESI Fig. S5.† This HRTEM image (Fig. S5†) provides information about the interplanar spacing of the lattice fringes. The interplanar spacing (*d*) value was measured to be 0.202 nm, which represents the (111) plane of the Ni crystals.

**Fig. 2:**
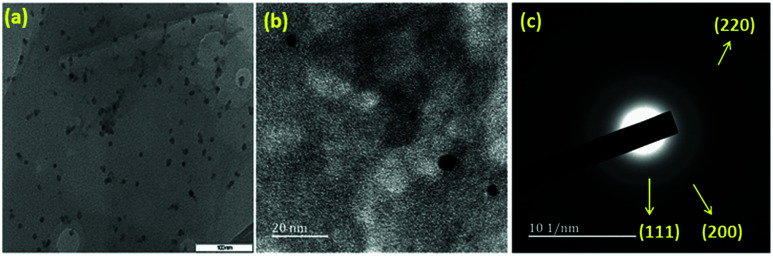
TEM image of (a) Ni(0)RGO (scale bar = 100 nm), (b) high magnification image of Ni(0)RGO (scale bar = 20 nm), and (c) electron diffraction pattern image of Ni(0)RGO.

C–C coupling reactions have been reported by many researchers under inappropriate conditions and using highly expensive mono-metals like Pd, Au, and Pt, and bimetal combinations like Pd–Pt, Pd–Au, and Pt–Au as catalysts. Molnár and Papp reported that a C–C cross-coupling reaction over a Pd-modified montmorillonite material at 150 °C gave 92% yield after reacting for 2–3 hours.^35^ Corral *et al.* reported that 35% conversion was achieved in the presence of organic solvent after 3 h.^36^ There are very few examples that use inexpensive metals, like Ni, as a catalyst for this C–C coupling reaction. Bhowmik *et al.* reported that a C–C coupling reaction with a Ni/RGO catalyst gave 91% yield at 60 °C after reacting for 4 h.^37^ Sengupta *et al.* reported that a Ni/RGO catalyst produced a C–S coupling product at 92% yield in 3 h at 100 °C.^38^ The inbuilt drawback of these studies is the need for either an organic solvent or a high temperature.

So, in this communication, we report Ni(0)-modified reduced graphene oxide as a catalyst to improve the yield of this coupling reaction under suitable and appropriate conditions. For the initial study, without the use of the catalyst, the C–C Kumada–Corriu cross-coupling reaction was examined using iodoanisole and phenyl magnesium chloride in the presence of a tetrahdrofuran medium at 60 °C; no methoxybiphenyl product was observed. The same reaction was run under similar conditions using the Ni(0)RGO catalyst, which produced an excellent yield (92%) of methoxybiphenyl (Table 1). Table 1 indicates that the C–C cross-coupling reaction with the RGO catalyst and Ni(ii)RGO showed a poorer performance compared to that conducted with the Ni(0)RGO catalyst.

**Table 1:** Catalytic activity of different catalysts toward the C–C cross-coupling reaction^a^

Catalyst	Time (h)	Temp. (°C)	Yield (%)
Without catalyst	5	60	—
RGO	5	60	9
Ni(ii)RGO	5	60	62
Ni(0)RGO	5	60	92

a 4-Iodoanisole (1 mmol), catalyst (0.1 mmol), PhMgCl (1.8 mmol), solvent (2 mL), under N_2_.

To assess the method, we carried out C–C coupling reactions with different substituents of aryl iodide moieties under similar conditions in the presence of the Ni(0)RGO catalyst, as detailed in Table 2. The product yields indicate that *para* iodobenzene gives a better yield than *ortho* iodobenzene. Generally, C–C coupling reactions proceed *via* three steps: oxidative addition, transmetallation, and reductive elimination.^39,40^ In the oxidative addition step, the organo halide reacts with Ni(0) to form a Ni(ii) complex. In the transmetallation step, the aryl group of the Grignard reagent reacts with the Ni(ii) complex to form an Ar–Ni–Ar–(O–CH_3_) complex and a magnesium iodide salt. In reductive elimination step, the coupling product is formed. Out of the two substituents, the *para* substituent is more favoured than the *ortho* substituent, due to steric hindrance.

**Table 2:** C–C cross-coupling reactions of different substituents of iodo-arenes with Grignard reagents in the presence of the catalyst, Ni(0)RGO^a^

Sl no.	Aryl halide (ArX)	Grignard reagent	Product	Yield (%)
1	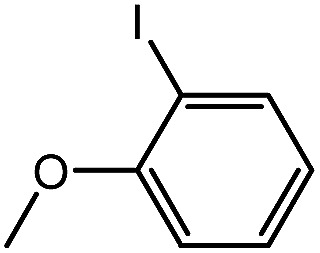	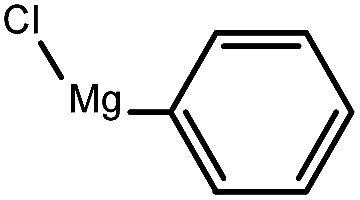	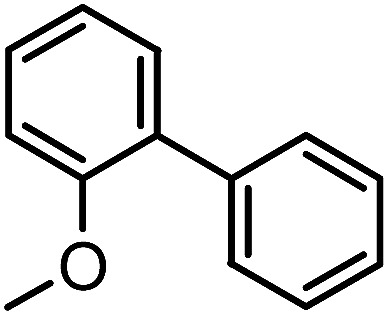	71
2	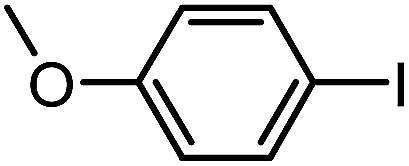	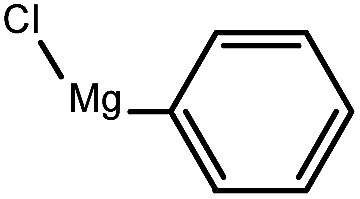	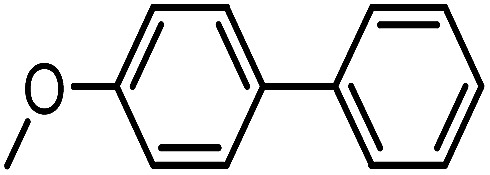	92

a Iodoanisole (1 mmol), catalyst (0.1 mmol), PhMgCl (1.8 mmol), solvent (2 mL), under N_2_, time (5 h), temperature (60 °C).

In order to optimize the reaction, we carried out the Kumada cross-coupling reaction at different temperatures under similar conditions (Fig. 3). The yield of the methoxybiphenyl coupling product increases from 78 to 92% with an increase in the temperature from room temperature to 60 °C. The yield of the coupling product did not increase with further increases in the temperature from 60 °C to 70 °C. Thus, the optimum reaction temperature is 60 °C for this coupling reaction using the Ni(0)RGO catalyst.

**Fig. 3:**
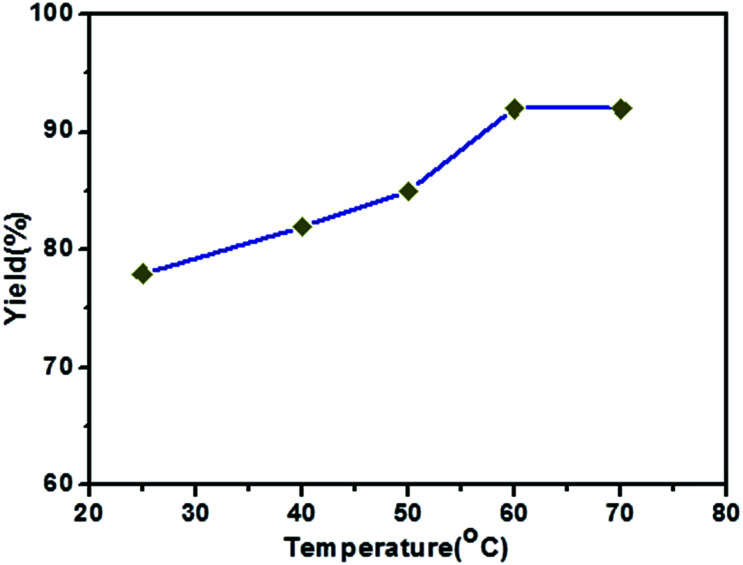
Effect of reaction temperature on the C–C Kumada–Corriu cross-coupling reaction when using the Ni(0)RGO catalyst.

The C–C Kumada–Corriu cross-coupling reaction performance of the Ni(0)@RGO catalyst was evaluated and compared with that of other Ni catalysts in ESI Table S1.† As can be seen from the data, the proposed Ni(0)@RGO catalyst proves to be superior, with a better yield compared to other methods using Ni/RGO-40 and NiCl_2_(dppf) catalysts.^40^ The heterogeneity test for the catalyst was carried out under the same reaction conditions. The coupling reaction was stopped after 3 h, the catalyst was filtered, and then the reaction was continued for the remainder of the reaction time (Fig. 4). In Fig. 4, the pink horizontal line shows the constant yield of the coupling product. Hence, no reaction occurred in the absence of the catalyst, and the reaction only happens in presence of heterogeneous catalyst composite.

**Fig. 4:**
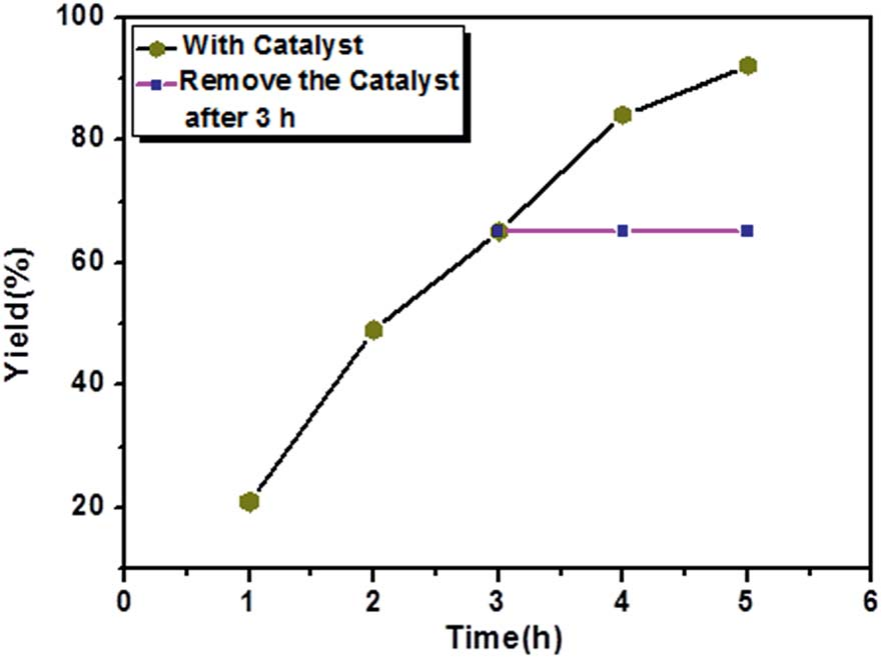
Heterogeneity test for Ni(0)RGO catalyst over C–C Kumada–Corriu cross-coupling reaction.

For commercialization purposes, the recovered catalyst was regenerated, followed by washing with solvent several times, and drying at the same temperature. The used catalyst was tested under the same reaction conditions several times (ESI Fig. S6†). The catalyst was active up to the 5^th^ cycle. In the 6^th^ cycle, the yield decreased by up to 13% due to leaching of the metal particles from the support surface.

In conclusion, Ni nanoparticles, with an average particle size of 12 nm, supported on reduced graphene oxide were successfully prepared by a simple procedure. The material demonstrated excellent catalytic activity (92% yield), good stability, and reusability for the Kumada–Corriu cross-coupling reaction. The absence of Ni(OH)_2_ and NiO particles and the presence of Ni(0) particles was confirmed by XRD analysis. TEM analysis confirmed that the Ni nanoparticles, with an average particle size of 11 nm, were uniformly distributed over the reduced graphene oxide surface. The catalyst also displayed good stability during recycling tests.

## Conflicts of interest

There are no conflicts to declare.

## Supplementary Material

NA-001-C8NA00245B-s001
