# Supplementary material for: Ni nanoparticle supported reduced graphene oxide as a highly active and durable heterogeneous material for coupling reactions
Source: Nanoscale Adv. 2019 Jan 29;1(4):1527–30. doi: 10.1039/c8na00245b (PMC9418947; doi:10.1039/c8na00245b)
Supplement: NA-001-C8NA00245B-s001 [file NA-001-C8NA00245B-s001.pdf]

Electronic Supplementary Material (ESI) for Nanoscale Advances.
This journal is © The Royal Society of Chemistry 2019

Supplementary information

Materials and Reagents:

Graphite powder (Sigma-Aldrich), H_2SO_4 (98%) CC Imelmann (Pty) Ltd.(Robertsham, South Africa), $NiCl_2$ (Acros). 4-Iodoanisole (98%), 3-Iodoanisole (99 %), Phenyl magnesium chloride solution, 4-Methoxybiphenyl (97%), 2-Methoxybiphenyl (99%) and Hydrazine hydrate were procured from Sigma-Aldrich. $KMnO_4$ (99%), $NaNO_3$ (99%), H_2O_2 (30%), were procured from BDH Chemicals Ltd. (South Africa), Radchem (South Africa), Glassworld and Chemical Suppliers CC (South Africa) respectively.

Materials Synthesis:

Graphite powder (1 g), $NaNO_3$ (0.5 g) and con. H_2SO_4 (23 ml) in a beaker and constant stirring with ice bath for 2 h. Then, the mixture was put on magnetic stirrer with hot plate at 40 °C and $KMnO_4$ (6 g) crystalline powder was added slowly and stirring continued for 30 min. Then, distilled water (50 ml) was added to the mixture and stirred for further 20 min, followed by addition of H_2O_2 (30 %) till the colour of the solution changed from dark brown to yellow. Upon the colour change, water (50 ml) was added to the resulting solution. Them, the solution was centrifuged, separated and filtered with double distilled water several times. The product was dried in a vacuum oven at 60 °C overnight to obtain GO

0.883 g of $NiCl_2$ and 20 ml of DD water are constant staring in a round bottom flask for 20 min. Then hydrazine hydrate (15 mL) was added to this solution and heated to 75 °C for 20 min with constant stirring. Then 1 g of graphene oxide was added in the aqueous solution to ultrasonication for 20 mins. Then, the aqueous suspension was added to the above mixture. Finally, the mixture was vigorously stirred at 80 °C for 4 h. The solid material was separated by washing with ethanol followed by water, then the material was dried in an air oven at 60 °C for 24 h.

Equipment and Methods:

The X-ray diffraction study was performed on a Bruker D8 Advance instrument with CuKα as a radiation source. The average crystallite size (d) was measured from XRD patterns using the Scherrer's equation (Equation 1):

$$d = k\lambda/\beta\cos\theta \qquad (1)$$

Where, k = 0.9, θ = Bragg's angle and β = full width at half maximum.

The Transmitted electron microscopy images were observed on a Jeol JEM-1010 electron microscope with iTEM software. Jeol JEM 2100 Electron Microscope was used for capturing High resolution of TEM images. The JEOL JSM-6100 microscope was used for both scanning electron microscopy and EDX measurements. Raman spectra are collected on a Perkin Elmer 1200 Raman spectra are collected on a DeltaNu advantage 532™ Raman Spectrometer.

General procedure for catalytic reaction:

1 mmol of 4-Iodoanisole, 2 ml of THF solvent and 0.1 mmol of the Ni catalyst were taking in a round three necked volumetric flask under nitrogen atmosphere. Then, the dropwise addition of PhMgCl (1.8 mmol in 1 ml of THF), in the reaction mixture. The reaction mixture was constantly stirred for 5h at 60 °C. After completion of the reaction, the reaction was filtered and the resulting mixture was analyzed by GC.

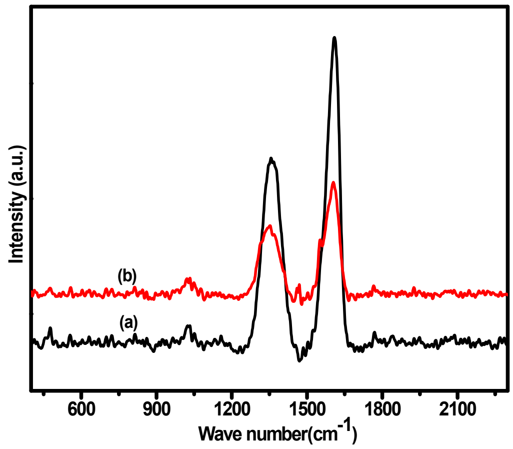

Figure S1: Raman spectra of GO (a) and Ni(0)RGO(b)catalyst.

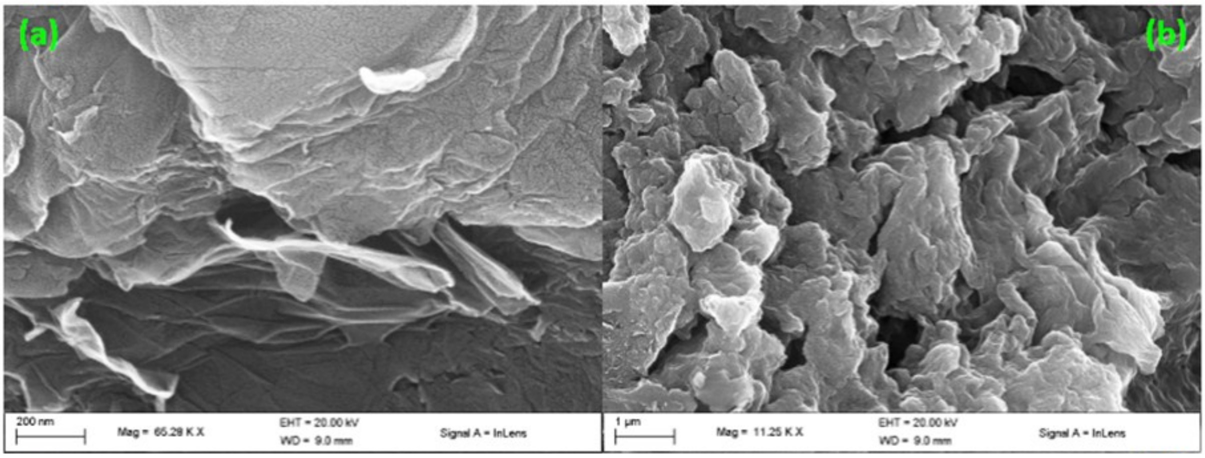

Figure S2: Scanning electron microscopy (SEM) image of GO (a) and Ni(0)RGO(b) catalyst.

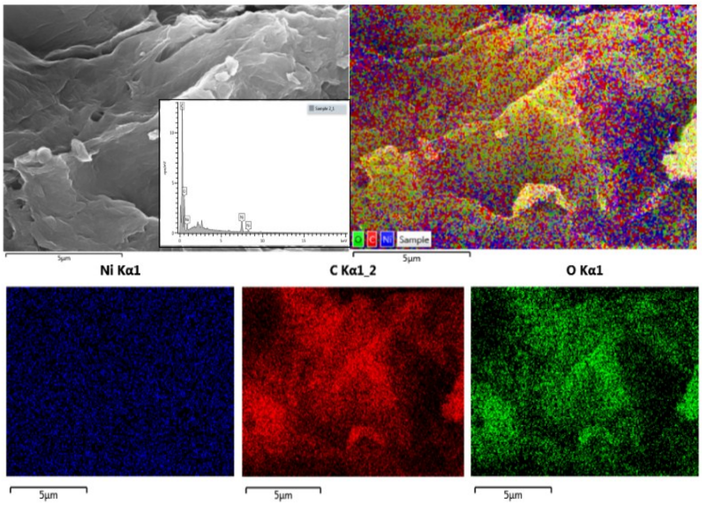

Figure S3: SEM-EDX image and color mapping image of Ni(0)RGO catalyst.

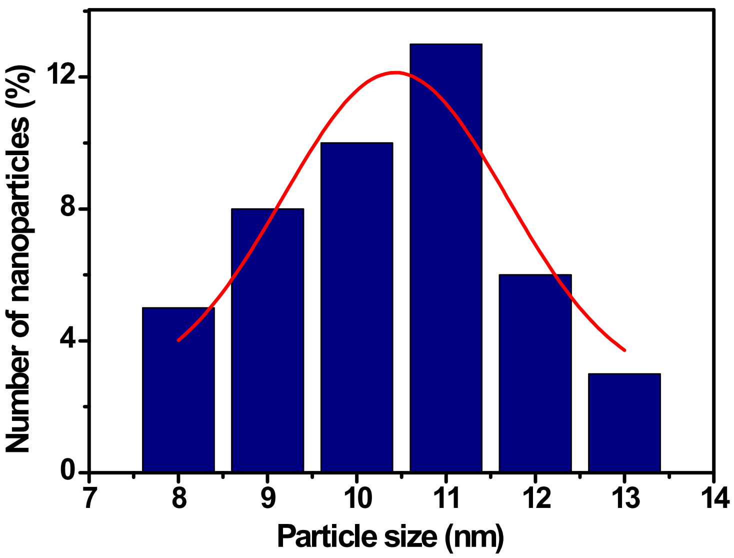

Figure S4: Particle size histogram for Ni nanoparticle of Ni(0)RGO catalyst.

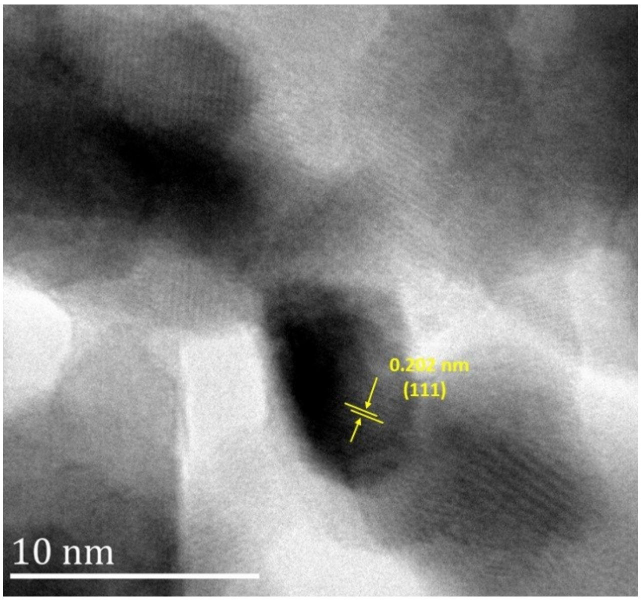

Figure S5: High-resolution transmission electron microscopy images of Ni(0)RGO catalyst

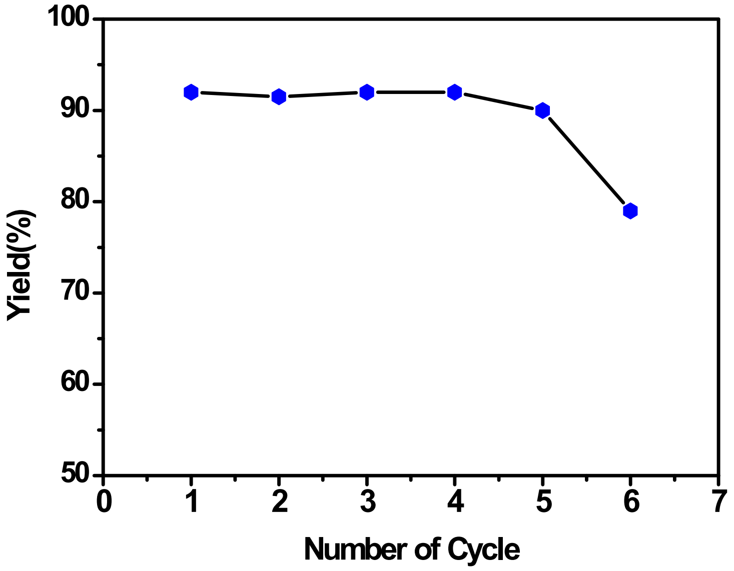

Figure S6: Reusability test over Ni(0)RGO catalyst in C-C cross-coupling reaction.

Table S1: Comparison study of C-C Kumada-Corriu cross coupling reaction catalysed by Ni catalysts.

Catalyst	Temperature	Yields	References
Ni/RGO-40	60	91	[37]
$NiCl_2$(dppf)	RT	60	[38]
Ni(0)@RGO	60	92	Present work
